# Multifactorial Screening Tool for Determining Fall Risk in Community-Dwelling Adults Aged 50 Years or Over (FallSensing): Protocol for a Prospective Study

**DOI:** 10.2196/10304

**Published:** 2018-08-02

**Authors:** Anabela Correia Martins, Juliana Moreira, Catarina Silva, Joana Silva, Cláudia Tonelo, Daniela Baltazar, Clara Rocha, Telmo Pereira, Inês Sousa

**Affiliations:** ^1^ Physiotherapy Department Coimbra Health School Polytechnic Institute of Coimbra Coimbra Portugal; ^2^ Fraunhofer Portugal AICOS Porto Portugal; ^3^ Sensing Future Technologies Coimbra Portugal; ^4^ Complementary Sciences Department Coimbra Health School Polytechnic Institute of Coimbra Coimbra Portugal; ^5^ Institute for Systems Engineering and Computers at Coimbra Coimbra Portugal; ^6^ Clinical Physiology Department Coimbra Health School Polytechnic Institute of Coimbra Coimbra Portugal

**Keywords:** accidental falls, primary prevention, adults, clinical protocol, pressure platform, inertial sensors

## Abstract

**Background:**

Falls are a major health problem among older adults. The risk of falling can be increased by polypharmacy, vision impairment, high blood pressure, environmental home hazards, fear of falling, and changes in the function of musculoskeletal and sensory systems that are associated with aging. Moreover, individuals who experienced previous falls are at higher risk. Nevertheless, falls can be prevented by screening for known risk factors.

**Objective:**

The objective of our study was to develop a multifactorial, instrumented, screening tool for fall risk, according to the key risk factors for falls, among Portuguese community-dwelling adults aged 50 years or over and to prospectively validate a risk prediction model for the risk of falling.

**Methods:**

This prospective study, following a convenience sample method, will recruit community-dwelling adults aged 50 years or over, who stand and walk independently with or without walking aids in parish councils, physical therapy clinics, senior’s universities, and other facilities in different regions of continental Portugal. The FallSensing screening tool is a technological solution for fall risk screening that includes software, a pressure platform, and 2 inertial sensors. The screening includes questions about demographic and anthropometric data, health and lifestyle behaviors, a detailed explanation about procedures to accomplish 6 functional tests (grip strength, Timed Up and Go, 30 seconds sit to stand, step test, 4-Stage Balance test “modified,” and 10-meter walking speed), 3 questionnaires concerning environmental home hazards, and an activity and participation profile related to mobility and self-efficacy for exercise.

**Results:**

The enrollment began in June 2016 and we anticipate study completion by the end of 2018.

**Conclusions:**

The FallSensing screening tool is a multifactorial and evidence-based assessment which identifies factors that contribute to fall risk. Establishing a risk prediction model will allow preventive strategies to be implemented, potentially decreasing fall rate.

**Registered Report Identifier:**

RR1-10.2196/10304

## Introduction

Falls and fall-related injuries are major health problems among older adults [1]. About a third of community-dwelling persons aged 65 years or older fall each year [1,2]. Although fall studies are mainly associated with this age group, persons aged 50 years or older often underestimate their risk of suffering a fall [3]. Moreover, fall-related injuries are the most common reason for hospital admission in those who are 50 years or older [4], and screening recommendations were already given to those identified as being at a higher risk of falling because of an underlying condition [1].

Falls are complex and have multifactorial etiologies [5,6]. Different factors can increase the risk of falling, particularly psychotropic medications and polypharmacy, and mitigation of these factors was found to reduce fall rates [7,8]. Vision impairment is also considered a fall risk factor inherent to changes in visual acuity, development of cataracts, macular degeneration, glaucoma, and other conditions related to the aging process [7]. High blood pressure and heart rate and rhythm abnormalities, such as carotid sinus hypersensitivity, vasovagal syndrome, bradyarrhythmias, and tachyarrhythmias, are similarly associated with falls. Environmental hazards at home related to lighting, chair and bed height, floor surfaces, and other factors create opportunities for falls and have been included as essential components of fall prevention programs [7,9]. Additionally, changes in musculoskeletal and sensory system functions that are associated with aging lead to deficits in maintaining postural stability [10]. In turn, fear of falling (FoF) can have a major impact on older adults, raising caution and restricting activities leading to physical fragility [11]. Finally, individuals who experienced previous falls and with multiple risk factors are at a higher risk of falling [12,13].

Falls can lead to minor injuries such as bruises, lacerations, or abrasions, and 10% of cases result in fractures [8,14], thus contributing to significant increases in morbidity and mortality [8,9]. Direct health care costs associated with this phenomenon are high [15], reaching 25 billion euros per year in the European Union [2].

The evidence shows that falls can be prevented by screening for risk factors and by the prescription of tailored interventions [16]. These results allow the professional to identify those in need of more detailed assessments [17]. The assessment of fall risk factors is the focus of a number of different screening methods, such as Morse Fall Scale [18], Berg Balance Scale [19], and Performance‐Oriented Assessment of Mobility Problems in Elderly Patients [20].

Recently, in addition to traditional methods, instrumentation has been integrated in some standard assessment tests, adding value to the existing methods because it gives additional quantitative information and eliminates the bias introduced by observation [21].

The aims of this study are to develop a multifactorial, instrumented, screening tool for fall risk based on functional tests, their metrics, and other potential risk factors and to prospectively validate a risk prediction model for the risk of falling among Portuguese community-dwelling adults aged 50 years or older.

## Methods

### Study Design

This study is a prospective longitudinal study, following a convenience sampling method.

### Setting

Individuals are voluntarily recruited from several settings within the community in different regions of continental Portugal, such as parish councils, physical therapy clinics, seniors’ universities, and other facilities.

Recruitment started in June 2016 and is ongoing. After the screening, the participants will receive monthly phone calls over a 12-month period to record the rate of falls.

### Participants

Inclusion criteria consist of adults aged 50 years or over, able to stand and walk independently with or without walking aids, and who are interested in participating in the study. Individuals will be excluded if they have severe sensorial impairments (deafness or blindness) or cognitive impairments, which preclude the ability to comprehend the questionnaires and functional tests included in the screening protocol.

### Ethical Considerations

Ethical approval was obtained from the Research Ethics Committee of Polytechnic Institute of Coimbra (Nº6/2017). All participants will give written informed consent before data collection begins as per the Declaration of Helsinki.

### Details of the Screening Protocol

The FallSensing screening tool is a technological solution for fall risk screening, which includes software, a pressure platform, and 2 inertial sensors.

#### Software

The software consists of a questionnaire that collects information about demographic and anthropometric data (age, sex, height, and weight), history of falls (previous 12 months, HoF), FoF, health conditions (heart attack, stroke, osteoarthritis, diabetes, Parkinson’s disease, osteoporosis, high blood pressure, high cholesterol, hearing and vision impairments, and urinary incontinence), medication, sedentary behaviors, upper extremities assistance needed to stand from a chair, living settings, alcohol habits, self-perceived health, and unintentional or involuntary weight lost as well as a detailed explanation about procedures to accomplish every functional test. Finally, 3 questionnaires concerning environmental home hazards, activities and participation profile related to mobility (PAPM), and self-efficacy for exercise are also included.

This app integrates a chronometer used to measure time for the Timed Up and Go (TUG) test, the 30 seconds sit to stand (30s STS) test, the step test, the 4-Stage Balance test “modified,” and the 10-meter walking speed test. In the 30s STS, the number of stands is registered manually by the physiotherapist. The same procedure will be used to register the number of steps. Additionally, the software records inertial sensors and pressure platform raw data. All the information will be available in a cloud server designed for the screening tool.

##### History of Falls

A fall can be defined as “an unexpected event, in which the participant comes to rest on the ground, floor, or lower level” and “excludes coming to rest against furniture, wall, or other structure” [1,8,22].

HoF is considered as a risk factor for falls [23] and the strongest single predictor of future falls [24] because it is associated with reduced lower limbs strength, gait, and balance impairments [12]. According to literature, older adults who have experienced one or more falls have 3 times the risk of falling again within the following year compared with those with no HoF [25]. Although the history and number of previous falls are self-reported, they are often used as golden standards in fall risk assessment studies [26].

HoF within the previous 12 months will be determined by self report, answering the question “Did you fall in the past 12 months? Yes-No.” If the participant has fallen, it will be asked if the fall was outdoor or indoor, the reason of the fall (slip, stumble, loss of consciousness, dizziness, lower extremities weakness, no special reason, and other), need of health services assistance, which health service (hospital, primary health care center), hospitalization (how many days), activity limitation and restrictions on participation (how many days), and fracture occurrences (wrist or hand, hip, skull or spine, and others).

##### History of Falls After 12 Months

Participants will be prospectively followed up for a 12-month period via monthly phone calls to record their fall occurrences. The fall rates will be recorded from the day of inclusion until voluntary dropout, loss of phone contact, or the end of the follow-up period (365 days later).

##### Fear of Falling

FoF is defined as “a lasting concern about falling that leads to an individual avoiding activities that he or she remains capable of performing” [27]. The literature states that FoF contributes to a loss of independence and disability through the restriction of activities [28,29] because it is associated with an increased risk of functional decline [30], reduced physical activity, lower perceived physical health status, lower quality of life, and increased institutionalization [31]. Despite being more frequent among fallers, FoF and activity restriction are not exclusive of these persons. In addition, FoF was significantly more frequent among women and among people living alone [32].

Considering the negative influence of FoF, its existence will be assessed by self report through the question “Are you afraid of falling? Yes-No.”

##### Health Conditions

There are certain conditions that can have a significant effect on fall rates in older adults, such as bladder incontinence, osteoarthritis, Parkinson’s disease, cardiovascular accidents, and conditions associated with cardiovascular disease, such as hypertension. Additionally, deficits in the somatosensory and vestibular systems can also contribute to falls because they are associated with an increase in postural sway, a strong indicator of standing balance [33].

The question “Do you have trouble seeing well or has it been more than 2 years since your eyes were last tested?” assesses changes in visual acuity, development of cataracts, macular degeneration, glaucoma, and other conditions related to the aging process that can contribute to increased risk of falling [7,33].

##### Medication

It is reported that approximately 20.3% of persons aged 55 years or over take 4 or more medicines [34]. Older adults taking more than 3 or 4 medicines were at an increased risk of recurrent falls [35]. Additionally, a significant association between falls and the use of sedatives and hypnotics, antidepressants, and benzodiazepines was found [34].

The number of medicines taken by each person was assessed by self report through the question “Do you take 4 or more different medicines per day? Yes-No.” The names of the medicines were also registered, and they were identified according to their pharmaceutical group (benzodiazepines, antidepressants, antipsychotics, antiinflammatory drugs, antihypertensive drugs, and others drugs).

##### Sedentary Behavior

Regular physical activity significantly decreases falls in older people; consequently, sedentary behaviors are associated with an increased incidence of falls [36,37]. To understand the community-dwelling adults’ sedentary behaviors using a self-reported question, we adopted the estimate measure of sedentariness calculated by Heseltine et al (2015), which is as follows: “Do you spend over 4 hours seated, 5 days or more per week?” This measure resulted from the analyses of sedentary behavior of a sample of 1104 adults aged 65 or more years, who answered the Physical Activity Scale for the Elderly. Additionally, older people underestimate sedentary behavior by self report by up to 50% [38].

##### Upper Extremities Assistance to Stand From a Chair

Upper extremities assistance to stand from a chair was assessed through the question “Do you need assistance from the upper extremities to stand up from a chair? Yes-No.” This action is an indicator of weak lower extremities muscles, a major reason for falling [39].

##### Living Settings

Because FoF is more frequent among older adults living alone [32], this protocol intends to assess the living settings through the question “Do you live alone? Yes-No.”

##### Alcohol Habits

Regular alcohol consumption among older adults has been linked to impaired balance and postural hypotension, which has been associated with frequent falls [40]. Furthermore, the intake of certain medications, such as benzodiazepines, even with small amounts of alcohol, can increase the risk of falling because of the interactions that can occur [41]. Participants are asked about their daily alcohol habits through the question “Do you drink alcohol every day? Yes-No.”

##### Self-Perceived Health

The self-perceived health (SPH) is considered a valid and reliable indicator of overall health status, a predictor of mortality and health services use. Several studies found an association with sociodemographic characteristics (such as sex, age, or education), chronic diseases, and functional status. Functional status, in particular, is recognized as a powerful determinant of SPH in older adults [42]. Older adults with FoF also demonstrated poor SPH [43]. SPH will be assessed by self report through the question “In general, do you perceive your health as excellent, very good, good, sufficient, or poor?”

##### Unintentional or Involuntary Weight Loss

Involuntary weight loss is one of the features that, simultaneously with others, can help to define a frailty phenotype [44]. Past literature reveals an association between the frailty phenotype and the number of previous falls in older people [45]. Therefore, participants will be asked if they had experienced a weight loss ≥4.5 kg or ≥5% of their body weight during the previous 12 months.

##### Functional Tests

###### Grip Strength

Hand grip strength is significantly correlated with lower limb muscular strength [46] and is a powerful predictor of disability, morbidity, and mortality [47]. This test will be performed with the participant seated on a standard chair without armrests [48], shoulder adducted and neutrally rotated, elbow flexed at 90 degrees, forearm neutral, wrist held between 0-15 degrees of ulnar deviation, and with the arm not supported [49]. A Jamar hydraulic hand dynamometer will be settled at the second handle position held with the dominant hand and during the performance of the test, it will be presented vertically in line with the forearm. The test is performed only once and the participant is encouraged to exert his or her maximal grip strength for 5 seconds [49,50]. The final score is measured in kilograms force (kgf). Normative data for this test are commonly analyzed by gender with males showing higher grip strength at all ages [51]. A score <15 kgf for females and <21 kgf for males identifies those at increased fall risk [52].

###### Timed Up and Go

The TUG test is used to assess dynamic balance during gait and transfers tasks, mobility, and lower body strength [53,54]. To perform this test, the person, wearing his or her regular footwear, is instructed to sit on a standard chair (chair height between 44 and 47 cm [55]) with his or her back against the chair back [49]. The person then stands up and walks straight for 3 meters as fast as possible, turns around, walks back, and sits down [54,56]. The person must stand up without help (cannot use the upper extremities for support); however, if a walking aid is needed, it should be placed next to the chair and can be used to perform the gait component of the test [54]. The test is performed only one time, the timing begins at the instruction “go” and stops when the patient sits on the chair [49]. A score of >10 seconds indicates which community-dwelling older adults are more likely to fall [53].

###### 30 seconds Sit to Stand

Lower body strength is important for maintaining functional capacity in older adults; therefore, its evaluation is critical [57-59]. The 30s STS test is a simple and effective instrument for assessing lower body strength and identifying muscle weakness in community-dwelling older adults and is one of the most important clinical functional evaluation tests [57,60]. The person is instructed to perform cycles of sit and stand up from a chair as many times as possible over 30 seconds [57,61].

The person starts the test seated in the middle of the chair (chair height between 40 and 43.3 cm), feet approximately shoulder-width apart and placed on the floor, and arms crossed by the wrists placed against the chest. The vocal instruction “go” indicates the test´s beginning and if the participant completes more than halfway up at the end of 30 seconds, it is counted as a full stand. The final score involves recording the number of stands a person can complete in 30 seconds [57,60]. The normative levels for the number of stands depend on age and gender [62].

###### Step Test

The step test was designed to assess dynamic standing balance and reproduce lower extremity motor control and coordination [63,64]. To perform the test, the person is asked to step on and off a block (7.5 cm height, 55 cm width, and 35 cm depth) placed against a wall as many times as possible for 15 seconds. The person should step onto the block with the whole foot and then return fully to the ground. The total number of completed steps in 15 seconds is recorded. The patient is unsupported and should look straight forward, although the test administrator must stand close by for safety. In cases where patients are overbalanced or need stabilization during the test, the counting of steps stops and the administrator records the complete number of steps prior to overbalancing [64-66]. This test is performed only for the dominant side, as indicated by the person being tested. A performance of <10 steps indicates a higher risk of falling [67].

###### 4-Stage Balance Test “Modified”

Deficits in balance can lead to falls and fall-related injuries, representing one of the most important intrinsic fall risk factors among older adults [68-70] that is commonly assessed in this population.

The 4-Stage Balance test “modified” evaluates balance. To complete this test, the person needs to progressively accomplish the following 4 different feet positions: side by side stance, semitandem stance (preferred foot forward with the instep of one foot touching the big toe of the other foot), tandem stance (one foot in front of the other, heel touching toe), and one legged stance (preferred leg for support, [71]).

The person is instructed to stand quietly on the pressure platform, arms along the body, with neither shoes nor assistive devices. The positions must be held for 10 seconds each without moving the feet, needing support, losing balance or touching the leg of support with the other leg [68,71] and must be performed with eyes open and then closed (excluding one legged stance position). The sequence will be side by side stance eyes open, side by side stance eyes closed, semitandem stance eyes open, semitandem stance eyes closed, tandem stance eyes open, tandem stance eyes closed, and one leg stand eyes open. If the person fails to accomplish one of the test positions, the test finishes. The final score will be the number of positions that are successfully completed. The inability to complete 10 seconds in the tandem stance position with eyes open has been associated with a higher risk of falling and mobility dysfunction [72,73].

###### 10-Meter Walking Speed

Walking speed is the product of a complex interaction of multiple body structures and functions, such as lower extremity strength, proactive and reactive postural control, motor control, and musculoskeletal condition [74,75]. Accessing gait speed (GS) as a screening tool can be useful for identifying those at risk or in need of intervention [75] because the gait speed results are related to various health outcomes, such as functional decline or FoF. Thus, GS can be a predictor of falls [74].

The performance of this test requires a 20-meter straight path with 5 meters for acceleration, 10 m for steady-state walking, and 5 meters for deceleration. Markers are placed at the 0-, 5-, 15-, and 20-meter positions of the path, and the time to walk along the 5 and 15 meters is registered [76]. The person is instructed to walk at his or her fastest walking speed wearing typical footwear and without running along the 20-meter path; an assistive device can be used if needed.

The range of normal walking speed is between 1.2 and 1.4 m/s because it varies by age, gender, and anthropometrics. A value of <0.4 m/s indicates the likely need for an assistive device at home; 0.4 to 0.8 m/s is correlated with limited mobility; 0.8 to 1.25 m/s indicates ambulation in the community with some risks; those with ≤1 m/s should start a program to reduce the risk of falling; and ≥1.42 m/s indicates a safe speed for crossing streets [74].

##### Questionnaires

###### Self-Efficacy for Exercise

Self-efficacy reflects the confidence that a person has to perform a certain behavior [77].

The self-efficacy for exercise is a 5-item scale intended to analyze the confidence that a person has to perform exercise according to 5 different emotional states, such as feeling worried or having problems, feeling depressed, feeling tired, feeling tense, and being busy.

Ratings are done using a 5-point Likert scale from 1 “not at all true” to 4 “completely true.” In between are 2, meaning “slightly true,” and 3, meaning “moderately true” [78].

###### Activities and Participation Profile Related to Mobility

PAPM is an 18-item scale intended to improve the understanding of the difficulties an individual experiences while performing certain daily activities in their natural environment. These activities can be conditioned by mobility and are related to the interactions and social relations, education, employment, money management, and social and community life and influence a person’s active participation in society [79]. Ratings are done using a 5-point Likert scale from 0, meaning “no limitation or restriction,” to 4, meaning “complete limitation or restriction.” In between, 1 indicates “mild limitation or restriction,” 2 indicates “moderate limitation or restriction,” and 3 indicates “severe limitation or restriction.” Because some activities may not apply, not all activities may be rated. As a result, an individual’s participation profile will be produced [79].

##### Home Safety Checklist for Fall Prevention

The Home Safety Checklist for Fall Prevention is a 38-item scale intended to identify home hazards in each room of a person’s home, namely the hallways, stairs, living or dining room, kitchen, bathroom, bedroom, and outdoors [80,81].

Ratings are assigned using a 3-point scale from 0 (indicating “no risk”), 1 (indicating “risk”), to 99 (indicating “do not apply”). A risk score is produced both to each room and for the home in general.

#### Pressure Platform (Universal Serial Bus Cable)

The PhysioSensing platform (Sensing Future Technologies, Lda) measures pressure distribution data, running at a frequency of 50 Hz. It consists of 1600 pressure sensors (10 mm by 10 mm) with a maximum value of 100 N/sensor. Voltage data are converted with an 8-bit A/D converter and is transmitted via Universal Serial Bus. Therefore, it is possible to receive raw data of each pressure sensor as well as the raw center of pressure coordinates (CoP) in cm. To obtain more precision in CoP displacements, an algorithm was employed to obtain CoP positions in millimeters using the matrix of pressure sensors [82]. The pressure platform collects valuable balance information during STS, the step test, and the 4-Stage Balance test “modified.” To collect useful information, participants should be barefoot.

#### Inertial Sensors (Bluetooth Connection)

Wearable inertial sensors were developed and assembled at Fraunhofer Portugal Research Center for Assistive Information and Communication Solutions, Portugal. Inertial data are collected from the built-in 3-axial accelerometer and 3-axis gyroscope, both sampled at 50 Hz. Raw data from the accelerometer are acquired for all the tests in m/s^2^ and raw data from the gyroscope are acquired in degrees/s^2^.

For the TUG test, 30s STS test, step test, 4-Stage Balance test “modified,” and 10-meter walking speed test, one inertial sensor is placed at the lower back, and one at the ankle. In the case of step test and 4-Stage Balance test “modified,” the ankle inertial sensor is placed on the dominant leg chose by the participant. Instrumentation with inertial sensors during the execution of standard tests gives additional quantitative information, such as the duration of the standing phase on TUG, contributing to better assessment, and characterizing a person’s mobility and balance conditions. Another advantage of using inertial sensors is that they eliminate the bias introduced by observation of movements and subjective assessment and the output extracted are potentially more reliable and reproducible [21].

### Statistical Analysis

Statistical analysis will be performed using IBM SPSS version 24 (SPSS Inc, Armonk, NY, USA) software. The sample size was calculated for an infinite population with a 95% confidence interval and a 5% margin of error to assess the number of participants needed to consider a representative sample of Portuguese population (minimum number of participants was 385). To perform the data analysis, the participants will be categorized as “fallers” (with one or more falls) and “nonfallers,” according to fall occurrences during the 12 month follow-up period.

The statistical approach will differ according to the level of measurement for the variables. The descriptive analysis will determine the mean and SD for the quantitative variables and frequencies for the qualitative ones. Differences in data between “fallers” and “nonfallers” will be analyzed by Student’s *t* test for independent samples or the chi-square test. Binary logistic regression analysis will be performed to determine a model that allows the prediction of falls from the functional tests and other variables. Receiver-operating characteristic (ROC) curve analysis will be used to identify the best cut-off score that distinguishes “fallers” from “nonfallers.” Sensitivity (percentage of “fallers” who were correctly identified), specificity (percentage of “nonfallers” that were correctly identified), and area under the receiver characteristic curve of the model will be calculated for prediction of falls. A significance of .05 will be considered for all comparisons, except for the quality of adjustment of the regression models, obtained with the Hosmer and Lemeshow test, whose significance is considered for *P*≥.05.

## Results

This prospective study is in progress. The enrollment has already begun and study completion is anticipated by the end of 2018. Permission letters for data collection were sent to the institutions identified as potential sites in the community to gather data. Authorization was obtained by their representatives and the screening procedures scheduled according to their daily routines. The results will be submitted to a leading journal for publication.

## Discussion

To develop a multifactorial screening tool to assess fall risk for community-dwelling persons, key risk factors for falls were identified. The occurrence of previous falls, visual impairment, urinary incontinence, and use of benzodiazepines are strong fall predictors [83]. In this protocol, different questions were included regarding the strongest predictors of falls. However, this screening protocol intended to collect more specific data which allow the characterization of each person. Different functional tests and questionnaires were included in the FallSensing screening protocol to accomplish a detailed evaluation of each case.

The FallSensing screening tool combines multiple validated instruments to identify multiple factors that influence fall risk. By understanding the major factors that increase fall risk, preventive strategies tailored for community-dwelling older adults can help decrease the fall rate and prevent fall-related injuries.

The lengthy screening time will probably represent a limitation of this protocol. However, one of the goals of this project is to prospectively validate a risk prediction model for fall risk by combining metrics collected by the pressure platform and inertial sensors complemented by additional collected data. The risk prediction model, defined according to ROC curve analysis, will allow to identify the most valuable data and consequently, shorten the protocol.

The National Institute for Health and Care Excellence recommends that the interventions to prevent falls should be patient-centered [1]; therefore, the stratification of risk will be a valuable tool for the FallSensing screening protocol, giving more detailed information and guiding the prescribing physiotherapist an intervention protocol toward fall prevention.

This investigation also will determine if the FallSensing screening protocol is feasible, valid, and acceptable in a Portuguese population.

The application of this prospective study protocol will allow us to understand the strengths and limitations of the FallSensing screening tool, leading to adaptations such as a shortened screening protocol.

## Abbreviations

CoP: center of pressure coordinates
FoF: fear of falling
GS: gait speed
HoF: history of falls
PAPM: activities and participation profile related to mobility
ROC: receiver-operating characteristic
SPH: self-perceived health
30STS: 30 seconds sit to stand
TUG: Timed Up and Go test
